# Social and therapeutic decline earlier than physical and psychological domains after discharge in heart failure patients: A patient-reported outcome measurements of latent transition analysis

**DOI:** 10.3389/fcvm.2022.965201

**Published:** 2022-09-20

**Authors:** Hong Yang, Jing Tian, Jing Li, Linai Han, Gangfei Han, Jinghua Zhao, Qinghua Han, Yanbo Zhang

**Affiliations:** ^1^Department of Health Statistics, Shanxi Medical University, Taiyuan, China; ^2^Department of Cardiology, The First Hospital of Shanxi Medical University, Taiyuan, China; ^3^Shanxi Provincial Key Laboratory of Major Diseases Risk Assessment, Taiyuan, China; ^4^School of Health Services and Management, Shanxi University of Chinese Medicine, Taiyuan, China

**Keywords:** chronic heart failure, latent class analysis, latent transition analysis, patient-reported outcomes measure, risk factors

## Abstract

**Background:**

Among patients with chronic heart failure (CHF), response shifts are common in assessing treatment effects. However, few studies focused on potential response shifts in these patients.

**Materials and methods:**

Data of CHF patient-reported outcome measures (PROMs) were obtained from three hospitals in Shanxi, China, from 2017 to 2019. A total of 497 patients were enrolled and followed up at 1 month and 6 months after discharge. Latent transition analysis (LTA) was employed to determine the longitudinal transition trajectories of latent subtypes in CHF patients in the physiological, psychological, social, and therapeutic domains.

**Results:**

The patients were divided into high- and low-level groups in the four domains according to the LTA. One month after discharge, the physiological and psychological domains improved, while the social and therapeutic domains remained unchanged. Six months after discharge, the former remained stable, but the latter deteriorated. The factors affecting the state transition in four domains were as follows. The influencing factor of the physiological domains are gender, age, tea consumption, smoking, alcohol consumption, physical activity, and light diet; those of the psychological domain are gender, occupation, smoking, alcohol consumption, and physical activity; those of the social domains are age; those of the therapeutic domains are education and income.

**Conclusion:**

The disease status of CHF patients has shifted over time. Risk factors accelerate the deterioration of patients’ condition. Furthermore, the risk factors of social and therapeutic domains deteriorate patients’ condition faster than those of physiological and psychological domains. Therefore, individualized intervention programs should be given for CHF patients who may be transferred to the low-level groups to maintain the treatment effect and improve the prognosis.

## Introduction

Chronic heart failure (CHF), a syndrome induced by cardiac dysfunction, is a leading cause of cardiovascular disease-related deaths. Approximately 26 million people suffer from CHF worldwide, and the number has been growing in recent years. Furthermore, CHF declines the quality of life of CHF patients and places increasingly heavy burdens on the affected families and the entire society (1–3). It has become a major threat to social progress and human health (4). Therefore, early assessment and management of patients with CHF are critical in primary care.

With the advances in diagnosis and treatment techniques in recent years, the survival of CHF patients has improved (5). However, only the cross-section data were considered in most classification criteria, while the longitudinal changes in the patients were neglected (6). The prolonged survival of HF patients could facilitate state shifts in assessing treatment effects. However, these state shifts were ignored in many studies (7, 8), leading to patients’ worsened condition and decreased quality of life (9).

Patient-reported outcomes measurements (PROMs) filled out by the patients accurately reflect their experiences and are important for clinical diagnoses and treatment. PROMs provide the indices of disease morbidity and prognostic assessment in clinical research and practice, thus having received wide attention (10). Tian et al. (11) devised PROMs for Chinese patients with CHF, which could fully reflect the state of the patients in the physiological, psychological, social, and therapeutic domains. CHF-PROMs facilitate the identification of different patient subgroups and the creation of longitudinal cohorts to analyze response shifts in different subgroups. However, the patient’s condition is a latent variable that cannot be directly observed. Therefore, latent transition modeling is required for subgroup classification and state transition analysis of CHF-PROMs data.

This paper aims to (1) explore the longitudinal dynamic development trend of CHF patient subtypes by analyzing the subtype characteristics of CHF patients and their disease conditions over time; (2) analyze the predictive effect of CHF patient subtype changes over time, gender, age, etc., on the change of subtype category.

## Materials and methods

### Study design and sample

Patient-reported outcome measures were obtained from inpatients diagnosed with CHF from May 2017 to January 2019 in three hospitals in Shanxi, China. Patients who met the inclusion criteria were required to complete the CHF-PROMs during hospitalization (baseline data T1). Patients were followed up in person or by telephone at 1 month (T2) and 6 months (T3) after discharge.

### Inclusion and exclusion criteria

Patients enrolled in the study must meet the following criteria. (1) Inpatients diagnosed with CHF (ICD-9 code 4289); (2) Patients at stages II–IV per the NYHA classification; (3) Patients over 18; (4) Patients voluntarily complete the questionnaire and are capable of writing. Furthermore, patients with cognitive impairment or psychiatric disorders and patients with acute cardiovascular events within 2 months were excluded.

### Measurements

This study was conducted using a questionnaire in Chinese, i.e., the CHF-PROMs. The questionnaire comprises 4 domains and 12 subdomains with 57 items, as shown in Table 1.

**TABLE 1:** Scale structure of chronic heart failure-patient-reported outcome measures (CHF-PROMs).

Domains	Subdomains	Items
Physical domain	Somatic symptoms (SOM)	**PHY1-, PHY2-, …, PHY7-, PHY8-**
	Appetite symptoms (APS)	**PHY9-, PHY10-, PHY11-, PHY12-**
	Independence (IND)	PHY13, PHY14, PHY15, PHY16
Psychological domain	Anxiety (ANX)	**PSY1-, PSY2-, …, PSY7-, PSY8-**
	Depression (DEP)	**PSY9-, PSY10-, …, PSY13-, PSY14-**
	Fear (FEA)	**PSY15-, PSY16-, PSY17-**
	Paranoid (PAR)	**PSY18-, PSY19-, PSY20-, PSY21-**
Social domain	Social support (SUP)	SOC1, SOC2, SOC3, SOC4, SOC5
	Support utilization (UTI)	SOC6, SOC7, SOC8
Therapeutic domain	Compliance (COM)	TRE1, TRE2, TRE3
	Satisfaction (SAT)	TRE4, TRE5, …, TRE9, TRE10
	Side effects of drugs (EOD)	**TRE11-, TRE12-**

“-” represent the reverse score of the item. PHY, physical domain; PSY, psychological domain; SOC, social domain; TRE, therapeutic domain.

Bold values indicates items are scored negatively.

The validity and reliability of CHF-PROMs were confirmed by reliability and validity verifications, and its clinical validity was confirmed by preliminary tests (11, 12). In this study, the Cronbach’s alpha of the data for 1-month follow-up after discharge was 0.858 and 0.810 for the physiological domain, 0.840 for the psychological domain, 0.689 for the social domain, and 0.791 for the therapeutic domain. The Cronbach’s alpha of the data for 6-month follow-up after discharge was 0.840, 0.767 for the physiological domain, 0.826 for the psychological domain, 0.652 for the social domain, and 0.793 for the therapeutic domain.

The scores of the items were calculated using a 5-point Likert scale. Due to the different number of items within each domain, the total score of each domain varies greatly. Therefore, the scores of each domain were converted into a 100 points system to facilitate the comparison between the results of each domain: (1) The scores in the CHF-PROMs were transformed from the range of 0–4 to the range of 1–5 according to whether the item was positively or negatively scored; (2) The transformed scores were added for each of the four dimensions; (3) The scores of each dimension were normalized to the range of 0–100 with the following formula:


$$S\,c\,o\,r\,e=\frac{T\,r\,a\,n\,s\,f\,o\,r\,m\,e\,d\,s\,c\,o\,r\,e-M\,i\,n\,i\,m\,u\,m\,p\,o\,s\,s\,i\,b\,l\,e\,s\,c\,o\,r\,e}{M\,a\,x\,i\,m\,u\,m\,p\,o\,s\,s\,i\,b\,l\,e\,s\,c\,o\,r\,e-M\,i\,n\,i\,m\,u\,m\,p\,o\,s\,s\,i\,b\,l\,e\,s\,c\,o\,r\,e}\times 100.$$

### Statistical analysis

Latent transition analysis (LTA) consists of two steps. The first step is to determine whether there are homogeneous subgroups within the CHF patient cohort during follow-up visits. Akaike information criterion (AIC) (13), Bayesian information criterion (BIC) (14), and adjusted BIC (aBIC) (15) were adopted to estimate the latent groups and models, where AIC, BIC, or aBIC with the minimum chi-square statistics was considered the proper latent groups with significant goodness of fit. The grouping accuracy was evaluated with the entropy that ranged from 0 to 1. The entropy is proportional to the grouping accuracy (16). Likelihood-ratio tests such as Lo–Mendell–Rubin (LMR) test and bootstrap likelihood ratio test (BLRT) were adopted to determine whether the difference between different grouping models was statistically significant (17, 18). The second step is to estimate the probability of temporal subgroup transition. AIC and BIC criteria were adopted to identify the correct model structure of the data, where >10% was regarded as a significant transition probability to identify the significant transition path during the tracking. MissForest was used to impute data with a missing ratio of less than 15% (19).

Latent transition analysis was conducted on Mplus 7.0. Furthermore, R 4.0.2 was adopted to give a statistical description, test common method variance, plot the probability density curves, and determine the subgroups’ thresholds. *P* < 0.05 was considered statistically significant.

## Results

**FIGURE 1:**
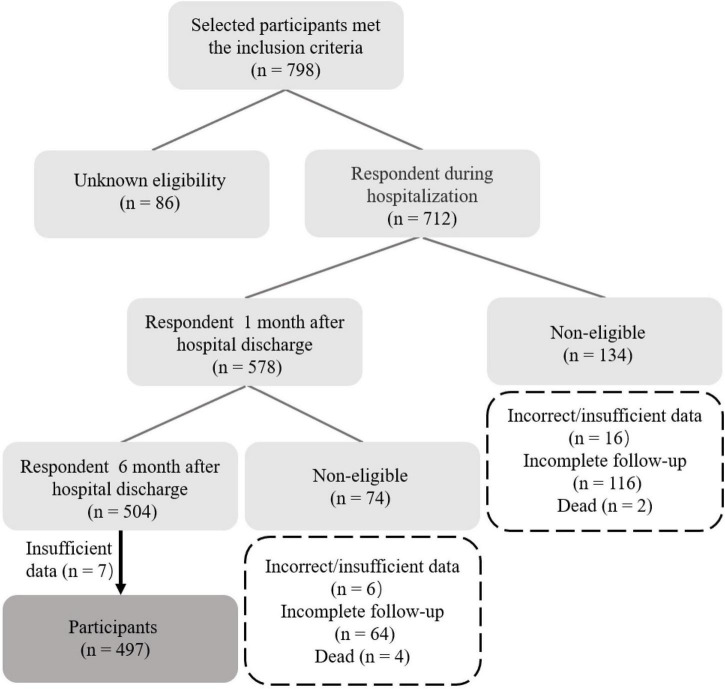
Flowchart of participant’s selection.

In this study, 712 questionnaires were distributed, of which 504 included two follow-up visits, and 497 were valid, with an effective rate of 98.6% (Figure 1). Among the 497 patients, the mean age was 67 ± 14.4 years, of which 280 (56.34%) were male. In this study, an unrotated principal component analysis was conducted using Harman’s single factor test on data from three time points to test the common method variance. The results indicated no excessive explanation of any of the factors for the data at three time points, suggesting no significant common method bias at three time points.

### Latent subgroup classification and transition

Supplementary Tables 1-1–1-4 show the latent profile model fitting indices and latent transition model fitting indices of 2–5 subtypes at T1, T2, and T3 time points in each domain. The final subgroups in each domain were classified according to AIC, BIC, aBIC, Entropy, MRT, BLRT, and latent class probabilities. Based on the analysis of the above evaluation indicators, patients were finally divided into high- and low-level groups in each of the four domains.

**FIGURE 2:**
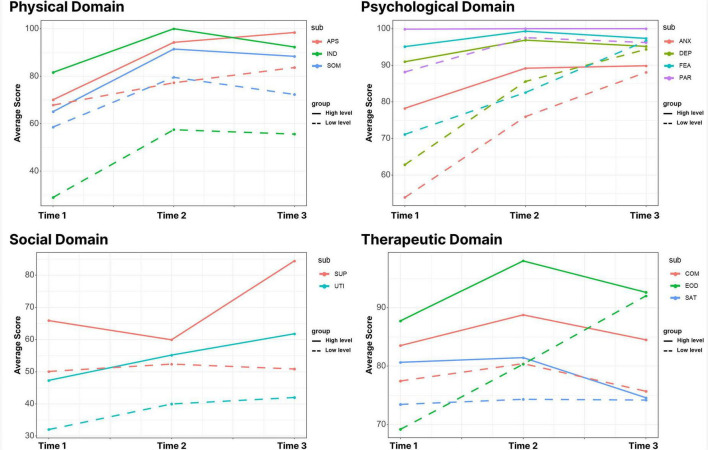
The average score distribution of people in different dimensions in the four domains of latent transition analysis (LTA). In the four domains, the mean distribution characteristics of the two subgroups at different time points after three potential shifts of dichotomy in different dimensions. SOM, somatic symptoms; APS, appetite symptoms; IND, independence; ANX, anxiety; DEP, depression; FEA, fear; PAR, paranoid; SUP, social support; UTI, support utilization; COM, compliance; SAT, satisfaction; EOD, side effects of drugs.

As shown in Figure 2, in the physiological domain, the difference between the scores of the two subgroups gradually decreases over time; in the psychological domain, the difference between the two subgroups in the four subdomains is more pronounced at the first two time points, but turns small at the T3; in the social domain, the difference between the two subgroups remains almost unchanged over time; in the therapeutical domain, the group with higher COM and SAT lower EOD, while the group with lower COM and SAT higher EOD. In addition, the difference between the two subgroups in the three subdomains was more pronounced at the first two time points but became small at T3.

Based on the subgroup classification, the CHF-PROMs score grading threshold of each subdomain was determined by plotting the probability density curves of the individuals in each subdomain and locating the intersection points of the curves. Therefore, the scores of patients in different subgroups could be preliminarily graded. Supplementary Figures 1–4 show the threshold scores for each subgroup of patients in each subdimension of each domain at the three time points.

**FIGURE 3:**
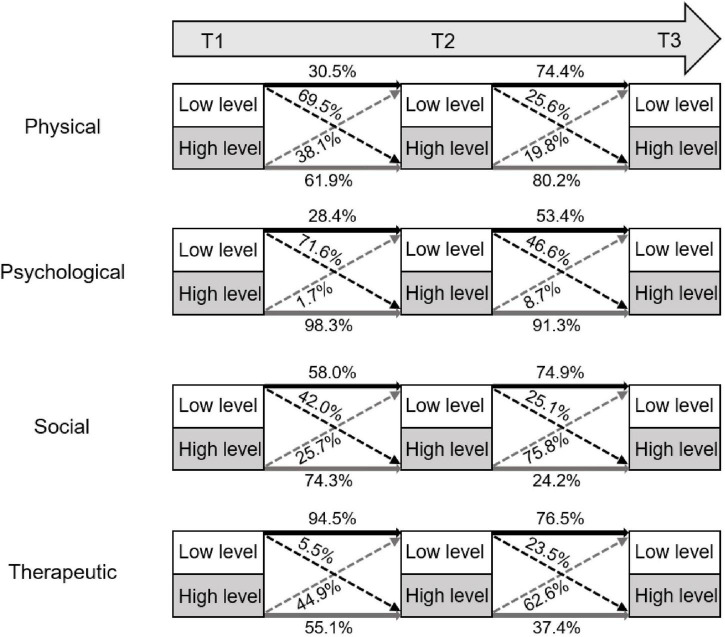
Latent transition trajectory of high and low levels in the four domains. It shows the state transitions over time in the different patient subgroups of the four domains.

In the physiological domain, there is an overall upward trend from T1 to T2, with patients in both the low and high levels having an over 50% probability of transitioning to the high level. In the psychological domain, there is an overall increasing trend from T1 to T2 as patients in the low-level group have a 71.6% probability of transitioning to the high-level group. However, patients in the high-level remained in their group from T2 to T3, and more patients transitioned to the low-level group compared with 3 months ago. In the social domain, patients in the high- and low-level groups have a 74.3 and 58% probability of remaining in their groups from T1 to T2, respectively, which are over 50%. However, there is an overall decreasing trend from T2 to T3 as patients in both groups have an over 70% probability of shifting to the low-level group. In the therapeutical domain, patients in the low-level group have an over 90% probability of remaining in their groups, and patients in the high-level group have a nearly 50% probability of transitioning to the low-level group. However, there is an overall decreasing trend from T2 to T3 as patients in both groups have an over 60% probability of remaining or transitioning to the low-level group (Figure 3).

The difference in covariate transition in different subgroups at different times was further explored (Supplementary Tables 1, 9, 15, 17).

### Covariate effects

As shown in Table 2, in the physiological domain, women, the elderly, and smoking patients are more likely to transition from the high-level to the low-level group. The probability of drinking patients transitioning from the high-level group is 1.12 and 0.46 times that of the non-drinking patients in the T1-T2 and T2-T3 phases, respectively. Patients with moderate tea consumption, moderate physical activity, and a light diet are more likely to transition from the low-level to the high-level groups.

**TABLE 2:** Odds ratios for covariates predicting transitions between latent statuses of different levels.

Domains	Covariates	Transition^a^ probabilities	Latent status^b^		Latent status^c^	
				
			Low level	High level	Low level	High level
Physical	Female	Low level	–	**1.65 (1.06–2.55)**	–	**2.96 (1.67–5.26)**
		High level	0.61 (0.30–1.22)	–	1.06 (0.54–2.01)	–
	The older age group (≥70)	Low level	–	**4.47 (2.82–7.09)**	–	**1.99 (1.13–3.51)**
		High level	**0.29 (0.13–0.62)**	–	0.62 (0.31–1.26)	–
	Tea habit	Low level	–	**0.52 (0.32–0.84)**	–	**0.34 (0.17–0.65)**
		High level	1.39 (0.67–2.90)	–	**2.37 (1.14–4.92)**	–
	Tobacco use	Low level	–	–	–	**1.84 (1.02–3.13)**
		High level	–	–	**0.61 (0.23–0.97)**	–
	Alcohol use	Low level	–	–	–	**0.46 (0.24–0.88)**
		High level	–	–	0.31 (0.86–1.11)	–
	Physical exercise	Low level	–	**0.24 (0.15–0.38)**	–	**0.25 (0.15–0.51)**
		High level	**6.95 (2.95–16.40)**	–	**12.71 (5.43–29.76)**	–
	Light diet	Low level	–	**0.22 (0.14–0.35)**	–	**0.28 (0.15–0.51)**
		High level	**13.85 (5.73–33.44)**	–	**4.78 (2.32–9.85)**	–
Psychological	Female	Low level	–	8.18 (0.95–70.80)	–	0.87 (0.45–1.71)
		High level	0.71 (0.34–1.48)	–	1.34 (0.41–4.43)	–
	Non-manual worker	Low level	–	0.53 (0.09–2.93)	–	–
		High level	0.83 (0.39–1.78)	–	–	–
	Tobacco use	Low level	–	5.73 (0.76–16.33)	–	–
		High level	0.57 (0.32–1.89)	–	–	–
	Alcohol use	Low level	–	–	–	0.94 (0.55–3.23)
		High level	–	–	1.19 (0.32–4.33)	–
	Physical exercise	Low level	–	2.14 (0.39–11.85)	–	**0.21 (0.09**–**0.45)**
		High level	**2.57 (1.14**–**5.78)**	–	**4.05 (1.21**–**13.54)**	–
Social	The older age group (≥70)	Low level	–	0.59 (0.33–1.08)	–	–
		High level	1.12 (0.67–1.86)	–	–	–
Therapeutic	High school degree above	Low level	–	0.58 (0.21–4.32)	–	0.61 (0.23–4.73)
		High level	**2.61 (1.31**–**5.73)**	–	1.06 (0.46–2.43)	–
	Middle and high income (>$1026)	Low level	–	0.52 (0.20–1.36)	–	**0.18 (0.04**–**0.83)**
		High level	**9.48 (4.27–21.03)**	–	1.45 (0.90–2.32)	–

All covariates entered simultaneously as predictors of latent status transitions. Covariate dichotomized into “yes/no” as reference class in logistic regressions. ^a^Rows for a reference time, ^b^Columns for T1 transfer to T2, ^c^Columns for T2 transfer to T3. The definition of each covariable is explained in the Supplementary material. There was no OR value for some variables because there was no statistical difference in the transfer of patients in different subgroups at different times. Bold values indicates the state transition is statistically significant.

In the psychological domain, female patients, patients not engaging in physical labor, smokers, and physically active patients are more likely to shift from the high-level to the low-level groups. Unlike the physiological domain, more drinking patients transition from the low-level to the high-level groups. Specifically, the probability of drinking patients transitioning from the low psychological level group is 2.81 and 1.19 times that of the non-drinking patients in the T1-T2 and T2-T3 phases, respectively.

In the social domain, more elderly patients shift from the low-level to the high-level groups. Specifically, the probability of elderly patients transitioning from the low-level group is 1.12 and 1.21 times that of the young patients in the T1-T2 and T2-T3 phases, respectively.

In the therapeutical domain, patients of high education and income levels are more likely to transition from the low-level to the high-level groups. In the T1-T2 phase, the probability of patients with high education levels transitioning from the low-level group is 2.61 times that of the patients with low education levels, and the probability of patients with high-income levels transitioning from the low therapeutical level group is 9.48 times that of the patients with low-income levels. In the T2-T3 phase, the probability of patients with high education levels transitioning from the low therapeutical level group is 1.06 times that of the patients with low education levels, and the probability of patients with high-income levels transitioning from the low therapeutical level group is 1.45 times that of the patients with low-income levels.

## Discussion

In this study, the transition of latent subtypes over time in CHF patients and its influencing factors were investigated based on the distribution of latent subtypes in CHF patients. LTA was adopted to study the state transition in the patients in the physiological, psychological, social, and therapeutic domains.

Patients with CHF were classified according to latent profile analysis (LPA), LPA-based LTA, and patient-reported outcomes. Specifically, the patients were classified into high- and low-level groups in the physiological, psychological, and social domains according to their subdomain scores. For instance, in the physiological domain, patients in the high-level group have a relatively better somatic condition, appetite and sleep, and physical independence, while patients in the low-level group have a relatively lower performance in the three subdomains. Patients were also classified into two groups in the therapeutical domain. The subdomain scores show that patients with high satisfaction and adherence are prone to drug side effects or to worry about the development of drug side effects. Studies on other diseases, such as psychiatric disorders and hypertension, have shown that medication side effects may affect patient adherence and satisfaction (20–22).

Latent transition analysis reveals that in the physiological domain, the high-level group is relatively stable in both phases, and the low-level group is more prone to state transition in the first phase and tends to stabilize in the second phase. Some studies on CHF focused on the effect of multiple factors on cardiac function classification or the prognosis of different cardiac function classes and ejection fractions but rarely touched on the physiological effect stratification (23–25). Patient state transition analysis in this study shows that patient states increase steadily before increasing at a decreasing rate. Therefore, additional attention should be given to patients with a transition from high to low levels. Multivariate analysis shows that gender, age, tea consumption, smoking, alcohol consumption, physical activity, and light diet statistically affect the patients. Female patients are more likely to transition to the low-level group in the physiological domain after discharge. Therefore, more attention should be paid to the prognostic changes in women’s physical states. Age is an important influencing factor in patient state. High age has been shown as a high-risk factor for poor prognosis in patients with acute heart failure (26), and extended care for elderly patients is a way to enhance the prognosis of patients (27).

In the psychological domain, most patients’ psychological level was improving, and the scores of the two groups were getting closer. The results indicate that although the patients’ states have changed, their overall psychological level is gradually improving. The change in patients’ psychological levels is similar to that in patients’ physical levels, probably because patients’ psychological levels are influenced by physiological changes (28). The probability and extent of state transition in mental health are greater than those in physical health, and the score analysis shows that an increase in patient scores cannot fully identify an improvement in the patient’s mental state (29). As mental health is a part of overall health, improving the patient’s psychological level helps to improve the patient’s physical condition (30). With the consideration of the normal psychological changes in patients and the actual scores, patient states should increase steadily before increasing at a decreasing rate. Therefore, patients transitioning from the high-level group to the low-level group (deteriorating patients) should be given additional attention. Analysis of psychological risk factors suggests that the statistically significant influencing factors are gender, occupation, smoking, alcohol consumption, and physical activity. Like the physiological domain, female patients are more prone to deterioration (31). Smoking is a risk factor for psychological state shifts in patients (32). Alcohol consumption has been shown to affect the patient’s psychological level positively. The conclusion is contrary to previous studies probably because moderate alcohol consumption can alleviate patients’ anxiety, and different levels of alcohol consumption have different effects on patients’ psychological and physical conditions. This study also suggests that physical exercise positively affects the patient’s psychological level, and prolonged exercise may be more effective in stabilizing patients’ emotional and psychological states. Different prognostic approaches should be taken to improve the psychological level of CHF patients at different stages and psychological levels.

In the social domain, patients’ social support and support utilization tend to be stable after discharge and begin to decline 1 month after discharge, which may be related to the improvement from the disease. However, decreased support does not mean reduced care for the patients. More attention should be paid to patients transitioning from the low- to the high-level groups (improving patients) to identify the factors for improvement and help patients with deteriorated and unimproved condition. Analysis of patient-specific changes in social levels shows that age is the only factor associated with changes in social levels, but the effect is small. Patients’ social support levels and support utilization correlate with patients’ psychological and physical states, and studies on other diseases suggest the influence of gender and age on the state shifts in the social domain (33).

In the therapeutical domain, the change in patients’ treatment experience levels is similar to that in the social domain, possibly due to the decreased adherence and satisfaction with the physician after discharge compared with the first discharge or the relaxed concern of the physician on patient’s treatment experience after discharge. Improved care, patient satisfaction, and adherence positively affect the patient (31). Therefore, patient management should be extended for a long period after discharge. Education level and income are found to positively affect the study of factors influencing changes in patients’ treatment experience. The reason for the result may be that patients with higher incomes and education levels are more likely to trust doctors and have sufficient financial resources to support adherence and satisfaction. Therefore, more attention should be paid to the treatment experience of low-income and low-education patients. Targeted out-of-hospital care and follow-up visits should be arranged, and care for their conditions should be given to improve their treatment experience, thus improving their health.

Studies on all four domains at these three time points show that post-hospital management of CHF patients is currently lacking. In the physiological and psychological domains, patient states improve shortly after discharge, but the trend of improvement slows down and stops after a long period. In the social and therapeutical domains, patients’ states maintain stable shortly after discharge but are more likely to deteriorate after a long period. Therefore, patients’ condition in social and therapeutical domains deteriorate faster than that in physiological and psychological domains, patients require more attention after discharge. Patient adherence and family support should be maintained to improve patient psychological levels. Furthermore, close attention should be paid to the conditions of CHF patients after discharge, and the treatments should be adjusted timely for different subtypes. For instance, patients with lower incomes and education levels should be treated with more patience and care, and treatment medication should be explained in more detail to improve their treatment experience. In addition to medication, more attention should be paid to lifestyle habits, such as moderate exercise, diet, and tea drinking, to improve patients’ long-term health conditions.

## Study limitations

This study revealed the trajectory of disease development and state transition in patients of different subtypes over time based on CHF-PROMs. The effects of different covariates, such as demographic characteristics and lifestyle, on longitudinal changes in patients’ quality of life, were compared to provide a basis for clinicians to develop personalized treatment plans according to their conditions and improve patients’ quality of life. However, there are still some limitations in this study. Some continuous variables were downgraded to categorical variables, which may cause information loss. Not all the covariates that could affect patient state transition were covered in this study, and the combined effect of multiple covariates was not studied. The research methodology should be further improved. In addition, the samples from three hospitals in Taiyuan cannot represent the whole Shanxi. The results of the study may have selection bias, and the extrapolation has certain limitations. The source data will be further optimized, the CHF sample size will be expanded, and more factors will be considered in the following studies. Furthermore, the combined effect of multiple factors and the intrinsic correlations between the four domains will be considered.

## Conclusion

This paper demonstrates post-discharge status transition in CHF patients based on longitudinal PROMs data. For CHF patients, more attention should be paid to the social and therapeutic areas after discharge, and the post-hospital management time should be appropriately extended. This study may provide additional knowledge and increase clinical understanding regarding the early assessment and management of patients with CHF.

## Data availability statement

The raw data supporting the conclusions of this article will be made available by the authors, without undue reservation.

## Ethics statement

The research program received medical and ethical approval from Shanxi Medical University (No. 2018LL128). The patients/participants provided their written informed consent to participate in this study.

## Author contributions

HY conceived the study, designed the study protocol, analyzed and interpreted the data, and drafted and wrote the manuscript. JT revised and reviewed the manuscript. JZ, JL, GH, and HY were responsible for collecting the data. HY and JZ participated in the data analysis. QH and YZ came up with the original concept for the study, oversaw the data analysis, and revised the manuscript. All authors contributed to the article and approved the submitted version.

## References

[1] Waheed A Lindenfeld J Albert N Boehmer J Heart Failure Society of America. HFSA 2010 comprehensive heart failure practice guideline. *J Card Fail.* (2010) 16:e1–194.20610207

[2] Soares-Miranda L Siscovick DS Psaty BM Longstreth WT Jr Mozaffarian D. Physical activity and risk of coronary heart disease and stroke in older adults: the cardiovascular health study. *Circulation.* (2016) 133:147–55. 10.1161/CIRCULATIONAHA.115.018323 26538582 PMC4814318

[3] Members WG Mozaffarian D Benjamin EJ Go AS Arnett DK Blaha MJ Executive summary: heart disease and stroke statistics–2016 update: a report from the American Heart Association. *Circulation.* (2016) 127:143–52.

[4] Zhou CJ Li AL Hou AH Zhang ZW Zhang ZX Dai PF Modeling methodology for early warning of chronic heart failure based on real medical big data. *Exp Syst Appl.* (2020) 151:113361. 10.1016/j.eswa.2020.113361

[5] Gupta A Ghimire G Hage FG. Guidelines in review: 2013 ACCF/AHA guideline for the management of heart failure. *J Nuclear Cardiol.* (2014) 21:397. 10.1007/s12350-013-9832-x 24343106

[6] Lichtenauer M Wernly B Paar V Rohm I Jung C Yilmaz A Specifics of fetuin-A levels in distinct types of chronic heart failure. *J Clin Lab Anal.* (2018) 32:e22179. 10.1002/jcla.22179 28213903 PMC6816921

[7] Romeyke T Noehammer E Stummer H. Patient-report-outcome-measure and incentives for inpatient chronic care in Germany. *Glob J Health Sci.* (2020) 12:127–43.

[8] Wohlfahrt P Zickmund SL Slager S Allen LA Nicolau JN Kfoury AG Provider perspectives on the feasibility and utility of routine patient-reported outcomes assessment in heart failure: a qualitative analysis. *J Am Heart Assoc.* (2020) 9:e013047. 10.1161/JAHA.119.013047 31937195 PMC7033831

[9] Gulek BG. Critical examination of current response shift methods and proposal for advancing new methods. *Qual Life Res.* (2021) 30:3325–42. 33595827 10.1007/s11136-020-02755-4 PMC8602164

[10] Yorkston K Baylor C. Patient-reported outcomes measures: an introduction for clinicians. *Perspect ASHA Spec Int Groups.* (2019) 4:1–8.

[11] Tian J Xue J Hu X Han Q Zhang Y. CHF-PROM: validation of a patient-reported outcome measure for patients with chronic heart failure. *Health Qual Life Outcomes.* (2018) 16:51. 10.1186/s12955-018-0874-2 29554963 PMC5859646

[12] Tian J Zhao J Zhang Q Ren J Han L Li J Assessment of chronic disease self-management in patients with chronic heart failure based on the MCID of patient-reported outcomes by the multilevel model. *BMC Cardiovasc Disord.* (2021) 21:58. 10.1186/s12872-021-01872-3 33516189 PMC7847136

[13] Akaike H. *Information Theory and an Extension of the Maximum Likelihood Principle.* New York, NY: Springer (1998).

[14] Schwarz G. Estimating the dimension of a model. *Ann Stat.* (1978) 6:461–4.

[15] Sclove SL. Application of model-selection criteria to some problems in multivariate analysis. *Psychometrika.* (1987) 52:333–43.

[16] Biernacki C Celeux G Govaert G. An improvement of the NEC criterion for assessing the number of clusters in a mixture model. *Pattern Recogn Lett.* (1999) 20:267–72.

[17] Yungtai L Mendell NR Rubin DB. Testing the number of components in a normal mixture. *Biometrika.* (2001) 88:767–78.

[18] Deb P Holmes AM. Estimates of use and costs of behavioural health care: a comparison of standard and finite mixture models. *Health Econ.* (2000) 9:475–89. 10.1002/1099-1050(200009)9:6<475::aid-hec544>3.0.co;2-h 10983002

[19] Stekhoven DJ Bühlmann P. MissForest—non-parametric missing value imputation for mixed-type data. *Bioinformatics.* (2012) 28:112–8. 22039212 10.1093/bioinformatics/btr597

[20] Richardson JL Marks G Levine A. The influence of symptoms of disease and side effects of treatment on compliance with cancer therapy. *J Clin Oncol.* (1988) 6:1746–52. 10.1200/JCO.1988.6.11.1746 3183704

[21] Li M Cai J Zhang P Fei C Xu F. Drug brand response and its impact on compliance and efficacy in depression patients. *Front Pharmacol.* (2016) 7:540. 10.3389/fphar.2016.00540 28119615 PMC5222824

[22] Lopez-Torres Lopez J Rabanales-Sotos J Lopez-Torres Hidalgo MR Milian Garcia RM Lopez Martinez C Blazquez Abellan G. Reliability and validity of the treatment satisfaction with medicines questionnaire (SATMED-Q) in persons with arterial hypertension. *Int J Environ Res Public Health.* (2021) 18:3212. 10.3390/ijerph18063212 33808854 PMC8003792

[23] Rashid M Curzen N Kinnaird T Lawson CA Myint PK Kontopantelis E Baseline risk, timing of invasive strategy and guideline compliance in NSTEMI: nationwide analysis from MINAP. *Int J Cardiol.* (2020) 301:7–13. 10.1016/j.ijcard.2019.11.146 31810815

[24] Huang CY Lin TT Wu YF Chiang FT Wu CK. Long-term prognostic value of estimated plasma volume in heart failure with preserved ejection fraction. *Sci Rep.* (2019) 9:14369. 10.1038/s41598-019-50427-2 31591412 PMC6779908

[25] Chen X Xin Y Hu W Zhao Y Zhang Z Zhou Y. Quality of life and outcomes in heart failure patients with ejection fractions in different ranges. *PLoS One.* (2019) 14:e0218983. 10.1371/journal.pone.0218983 31247042 PMC6597164

[26] Bettencourt P Rodrigues P Moreira H Marques P Lourenco P. Long-term prognosis after acute heart failure: a differential impact of age in different age strata. *J Cardiovasc Med.* (2017) 18:845–50. 10.2459/JCM.0000000000000507 28212137

[27] Jiang X Yao J You JH. Telemonitoring versus usual care for elderly patients with hospital discharge for heart failure in US: a cost-effectiveness analysis. *JMIR Mhealth Uhealth.* (2020) 8:e17846. 10.2196/17846 PMC7381019 32407288

[28] Sethares KA Viveiros JD Ayotte B. Uncertainty levels differ by physical heart failure symptom cluster. *Appl Nurs Res.* (2021) 60:151435. 10.1016/j.apnr.2021.151435 34247783

[29] Verdam MGE Oort FJ Sprangers MAG. Using structural equation modeling to investigate change and response shift in patient-reported outcomes: practical considerations and recommendations. *Qual Life Res.* (2021) 30:1293–304. 10.1007/s11136-020-02742-9 33550541 PMC8068637

[30] Wykes T Csipke E Williams P Koeser L Nash S Rose D Improving patient experiences of mental health inpatient care: a randomised controlled trial. *Psychol Med.* (2018) 48:488–97. 10.1017/S003329171700188X 28726599 PMC5757411

[31] Karaca A Durna Z. Patient satisfaction with the quality of nursing care. *Nurs Open.* (2019) 6:535–45. 10.1002/nop2.237 30918704 PMC6419107

[32] Taylor GMJ Baker AL Fox N Kessler DS Aveyard P Munafo MR. Addressing concerns about smoking cessation and mental health: theoretical review and practical guide for healthcare professionals. *BJPsych Adv.* (2021) 27:85–95. 10.1192/bja.2020.52 34513007 PMC7611646

[33] Shao Y Liang L Shi L Wan C Yu S. The Effect of social support on glycemic control in patients with type 2 diabetes mellitus: the mediating roles of self-efficacy and adherence. *J Diabetes Res.* (2017) 2017:2804178. 10.1155/2017/2804178 28626769 PMC5463190

