# Supplementary material for: Social and therapeutic decline earlier than physical and psychological domains after discharge in heart failure patients: A patient-reported outcome measurements of latent transition analysis
Source: Front Cardiovasc Med. 2022 Sep 20;9:965201. doi: 10.3389/fcvm.2022.965201 (PMC9530707; doi:10.3389/fcvm.2022.965201)
Supplement: Supplementary file 1 [file Data_Sheet_1.pdf]

Supplementary Material

Group definition:

T1-T2: It means transfer from hospitalization to 1 month after discharge.

T2-T3: It means transfer from 1 month after discharge to 6 months after discharge.

Deteriorate: It means that the patient has transferred from the high-level group to the low-level group, and the state of the field has deteriorated.

Improve: It means that the patient has transferred from the low-level group to the high-level group, and the state of the field has improved.

Low level persistence: It means that the patient has not metastasized and maintained the state of the previous stage, still at a low level.

High level persistence: It means that the patient has not metastasized and maintained the state of the previous stage, still at a high level.

Variable description:

According to the preliminary results of the research group [1], the age was divided into the younger age group (<70) and the older age group (>=70).

The education attainment was divided into high education level group (High School Degree Above) and low education level group.

Divided into manual worker group (Workers, farmers, etc.) and non-manual worker group (Teachers, physicians, office staff, etc.) according to occupational attributes.

According to the World Bank's 2019 definition of income level, we divided people into low (<=$1025), middle ($1026-$12375) and high income (>$12375) levels [2].

We defined at least once a month of 40 minutes of continuous exercise as the group with physical exercise, otherwise, as none [3].

We define the patients who eat more vegetables (7 times a week or more), eat less salt and oil as the light diet group, otherwise they are not [4].

Latent subgroup classification

As shown in Table S1-1, in the LCA part, the T1 results show that the AIC, BIC, and aBIC of the models with different numbers of categories have little difference, and the results of the two-category and four-category were better than the Entropy value, MRT, and BLRT. At the same T2 and T3 time points, the better models were three-category and two-category, respectively. The probability of each category should not be too low (<10%) after classification; otherwise, the number of patients in a certain category will be small. Therefore, T2 and T3 were finally determined as two categories. Although the category probability of T1 was relatively balanced due to the four categories, category two was finally determined with the consideration of the actual meaning of the model and the combination of the results of T2 and T3.

In LTA, the BIC of the second classification was better than that of the third classification, and the number of people with each transfer possibility was not less than 15. In order to facilitate the study of patient characteristics, combined with the results of LCA, the patient population was finally divided into two categories according to the three dimensions of the physiological field.In the LTA part, the BIC of the second classification is better than the third classification, and the number of people with each transfer possibility is not less than 15. In order to facilitate the study of patient characteristics, combined with the results of LCA, the patient population was finally divided into two categories according to the three dimensions of the physiological field.

Table S1-1 Fitting results of different classification LCA and LTA in the physical domain

Number of latent classes	AIC	BIC	aBIC	Entropy	LMR	BLRT	latent class probabilities
**LCA**							
**T1**							
2	13419.09	13461.18	13429.44	0.711	<0.0001	<0.0001	28.97/71.03

3	13399.72	13458.64	13414.20	0.767	0.0545	<0.0001	
4	13320.05	13395.81	13338.67	0.758	0.0006	<0.0001	19.12/20.43/13.28/27.16
5	13309.29	13401.88	13332.05	0.739	0.1401	<0.0001	
**T2**							
2	12163.123	12205.208	12173.468	0.923	0.0050	<0.0001	14.89/85.11
3	12027.874	12086.794	12042.357	0.934	0.0004	<0.0001	7.85/20.12/72.03
4	11901.847	11977.602	11920.469	0.923	0.0086	<0.0001	7.45/25.96/8.65/57.95
5	11857.682	11950.271	11880.442	0.905	0.0771	<0.0001	
**T3**							
2	11670.052	11712.137	11680.397	0.919	0.0310	<0.0001	16.70/83.80
3	11592.448	11651.368	11606.932	0.924	0.5263	<0.0001	
4	11499.115	11574.870	11517.737	0.920	0.4069	<0.0001	
5	11414.007	11506.596	11436.767	0.921	0.1313	<0.0001	
**LTA**							
2	36751.51	37113.45	36840.48	0.871			
3	36308.97	37428.45	36584.16	0.882			

In the LCA part shown in Table S1-2, the T1 results show that the AIC, BIC, and aBIC of the models with different numbers of categories have little difference, and the two-category results were better than the Entropy value and MRT and BLRT. At T2 and T3 time points, due to the insignificant difference in the fitting indicators of different categories and excessive grouping, the probability of some categories will be too small, and the number of patients belonging to one certain category will be too small. Therefore, based on the T1 results and the actual meaning of the model, they were classified as two categories.

In LTA, only two or three categories were considered because the number of potentially metastatic groups was large, but the number of patients was small. The BIC of the two classification was better than that of the third classification, and other indicators did not change much. Based on the results of LCA, the patient population

was finally divided into two categories according to the four dimensions of the psychological field.

Table S1-2 Fitting results of different classification LCA and LTA in the psychological domain

Number of latent classes	AIC	BIC	aBIC	Entropy	LMR	BLRT	latent class probabilities
**LCA**							
**T1**							
2	16252.10	16306.81	16265.55	0.933	0.0024	<0.0001	15.87/84.13
3	15942.41	16018.17	15961.03	0.925	0.0595	<0.0001	
4	15711.39	15808.19	15735.18	0.963	0.4976	<0.0001	
5	15587.10	15704.94	15616.07	0.926	0.1444	<0.0001	
**T2**							
2	12234.021	12288.733	12247.470	1.000	0.4630	<0.0001	0.81/99.20
3	11721.064	11796.819	11739.686	0.992	0.8977	<0.0001	0.81/4.83/94.37
4	11143.570	11240.367	11167.364	0.998	0.8774	<0.0001	0.60/0.81/5.03/93.56
5	10969.811	11087.652	10998.779	0.918	0.9458	<0.0001	0.60/0.81/5.03/21.13/72.44
**T3**							
2	11931.639	11986.350	11945.088	0.999	0.7612	<0.0001	2.21/97.79
3	11603.076	11678.831	11621.698	1.000	0.7876	<0.0001	1.41/13.68/84.91
4	11398.229	11495.026	11422.023	1.000	0.8317	<0.0001	1.41/1.41/13.48/83.70
5	10597.719	10715.560	10626.687	1.000	0.6519	<0.0001	0.20/1.41/3.02/11.67/83.70
**LTA**							
2	38912.02	39387.59	39028.93	0.955			
3	37018.60	38491.61	37380.70	0.981			

As shown in Table S1-3, in LCA, the T1 results show that the AIC, BIC, and aBIC of the models with the different number of categories have little difference, and the results of four categories are better obtained according to the Entropy value, MRT, and BLRT. At the same T2 and T3 time points, the better models were two-category and four-category, respectively. The probability of each category should not be too low (<10%) after classification; otherwise, the number of patients in a certain category will be small. Therefore, T1, T2, and T3 were finally determined as two categories.

In LTA, only two or three categories was considered because the number of potential metastatic groups was large, but the number of patients was small. The BIC of the second classification was better than that of the third classification. Although the Entropy value of the third classification was high, the number of components after the third classification was highly unbalanced. The two dimensions of the social domain fell into two categories.

Table S1-3 Fitting results of different classification LCA and LTA in the social domain

Number of latent classes	AIC	BIC	aBIC	Entropy	LMR	BLRT	latent class probabilities
**LCA**							
**T1**							
2	9072.496	9101.956	9079.737	0.628	<0.0001	<0.0001	33.60/66.40
3	9054.585	9096.671	9064.93	0.722	<0.0001	<0.0001	6.04/42.05/51.91
4	9043.654	9098.366	9057.104	0.757	0.4778	<0.0001	
5	8976.805	9044.143	8993.358	0.902	<0.0001	<0.0001	5.63/14.09/17.51/32.60/30.18
**T2**							
2	8444.726	8474.186	8451.968	0.616	0.0143	<0.0001	28.97/71.03
3	8438.205	8480.291	8448.550	0.728	0.1634	<0.0001	
4	8417.790	8472.502	8431.239	0.631	0.1007	<0.0001	

5	8399.084	8466.421	8415.637	0.701	0.0926	<0.0001	
**T3**							
2	8238.502	8267.962	8245.744	0.755	0.0141	<0.0001	14.29/85.71
3	8202.331	8244.417	8212.677	0.842	0.0067	<0.0001	2.21/17.10/80.68
4	8155.891	8210.603	8169.340	0.894	0.1019	<0.0001	
5	8136.174	8203.512	8152.727	0.856	0.0369	<0.0001	3.62/9.66/10.66/14.09/61.97
**LTA**							
2	25373.29	25621.59	25434.33	0.788			
3	25137.82	25903.78	25326.11	0.852			

As shown in Table S1-4, in the LCA part, the T1 results show that the AIC, BIC, and aBIC of the models with different numbers of categories are not much different, and the results of the four categories are better based on the Entropy value, MRT, and BLRT. At the same T2 and T3 time points, four-class and three-class models showed good performance, respectively. The probability of each category should not be too low (<10%) after classification; otherwise, the number of patients in a certain category will be small. Therefore, T1, T2, and T3 were finally determined as two categories.

In the LTA part, only two or three categories was considered because the number of potential metastatic groups was large, but the number of patients was small. The BIC and Entropy values of the two categories were greater than those of the three categories. Furthermore, combined with the results of the underlying profiles, the patient population was finally divided into two categories according to the three dimensions of the therapeutic area.

Table S1-4 Fitting results of different classification LCA and LTA in therapeutic domain

Number of latent classes	AIC	BIC	aBIC	Entropy	LMR	BLRT	latent class probabilities
**LCA**							

**T1**							
2	13092.86	13134.94	13103.2	0.914	0.0003	<0.0001	17.71/82.29
3	12967.83	13026.75	12982.32	0.922	0.0054	<0.0001	6.44/17.71/75.86
4	12836.58	12912.33	12855.2	0.981	0.0006	<0.0001	6.44/8.85/17.10/67.61
5	12746.05	12838.64	12768.81	0.978	0.0003	<0.0001	6.44/8.45/11.07/12.48/61.57
**T2**							
2	11455.632	11497.718	11465.977	0.967	0.0622	<0.0001	15.49/84.51
3	11275.906	11334.826	11290.390	0.973	<0.0001	<0.0001	8.65/11.67/79.68
4	11068.663	11144.417	11087.285	0.977	0.0159	<0.0001	8.25/10.26/12.27/69.22
5	11089.480	11182.069	11112.240	0.964	0.4288	<0.0001	
**T3**							
2	11220.078	11262.164	11230.423	0.982	0.1777	<0.0001	7.76/92.35
3	10910.075	10968.995	10924.559	0.996	0.0218	<0.0001	3.42/7.65/88.93
4	10659.410	10735.164	10678.032	0.980	0.7792	<0.0001	
5	10545.041	10637.630	10567.801	0.959	0.1687	<0.0001	
**LTA**							
2	35086.87	35448.8	35175.84	0.937			
3	34507.5	35626.98	34782.69	0.917			

Difference of covariate transition in different subgroups at different time

In the physical domain, the covariates statistically significantly ($P < 0.05$) influencing the transition between different subgroups from T1 to T2 include gender, age, tea-drinking, physical activity, and light diet, with chi-square being 10.327, 61.391, 8.675, 60.193, and 85.975, respectively. The covariates statistically significantly ($P < 0.05$) influencing the transition between different subgroups from T2 to T3 include gender, age, smoking, alcohol consumption, tea-drinking, physical activity, and light diet, with chi-square being 20.426, 56.628, 13.696, 18.402, 29.043, 87.627, and 46.741, respectively (Supplementary Table S1).

In the psychological domain, the covariates statistically significantly ($P < 0.05$) influencing the transition between different subgroups from T1 to T2 include gender, occupation, smoking, and physical activity, with chi-square being 18.114, 10.987, 9.411, and 10.158, respectively. The covariates statistically significantly ($P < 0.05$) influencing the transition between different subgroups from T2 to T3 include gender, alcohol consumption, and physical activity, with chi-square being 9.821, 18.739, and 24.345, respectively (Supplementary Table S9).

In the social domain, the covariate statistically significant ($P < 0.01$) influencing the transition between different subgroups from T1 to T2 is age, with chi-square being 8.732 (Supplementary Table S15).

In the therapeutical domain, the covariates statistically significantly ($P < 0.01$) influencing the transition between different subgroups from T1 to T2 and from T2 to T3 include education and income (supplementary Table S17).

PART I: Physical domain

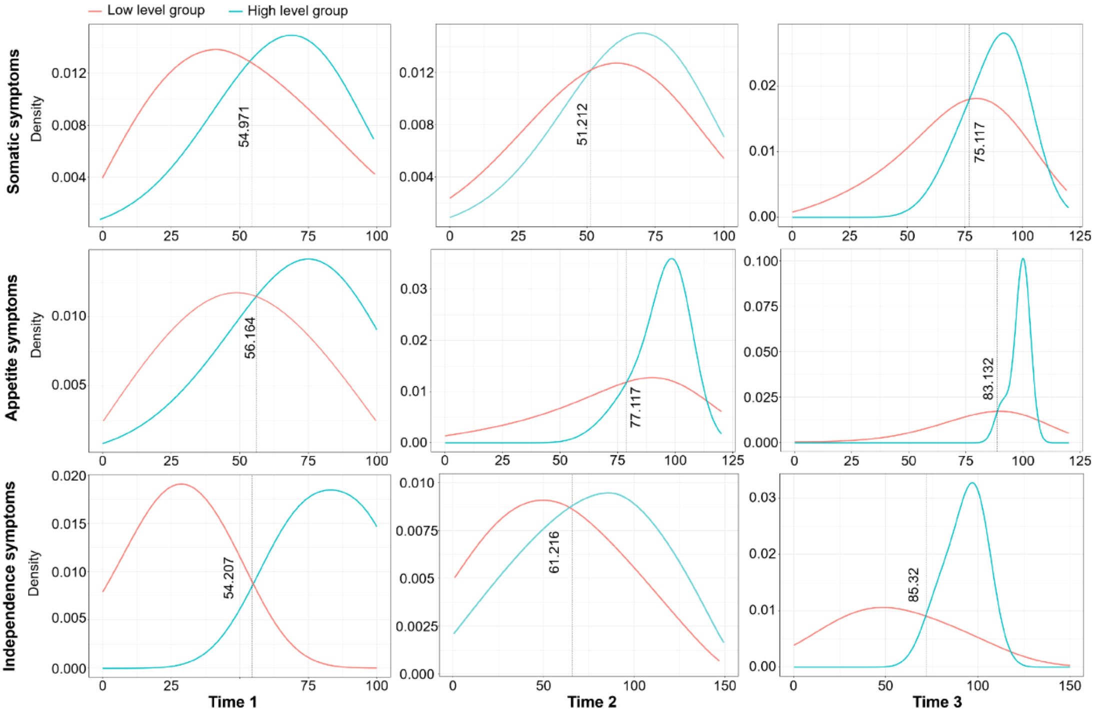

Figure S1. kernel density curves of two potential classes in the physiological domain

Table S1. Latent transfer distribution of covariates in the physical domain

Covariates	N (%)	Deteriorate	Improve	Low level persistence	High level persistence	χ^2
**T1-T2**						
**Gender**						
Male	280(56.34%)	69(51.49%)	53(54.08%)	20(41.67%)	138(63.59%)	**10.327***
Female	217(43.66%)	65(48.51%)	45(45.92%)	28(58.33%)	79(36.41%)	
**Age**						
<70	259(52.11%)	46(34.30%)	50(51.00%)	11(22.90%)	152(70.00%)	**61.391***
>=70	238(47.88%)	88(65.70%)	48(49.00%)	37(77.10%)	65(30.00%)	
**Educational attainment**						
High school degree above	322(64.70%)	88(65.70%)	63(64.29%)	36(75.00%)	135(62.22%)	2.882
Other	175(35.20%)	46(34.30%)	35(35.71%)	12(25.00%)	82(37.78%)	

**Career**						
Manual worker	278(55.93%)	77(57.46%)	51(52.04%)	33(68.75%)	117(53.92%)	4.287
Non-manual worker	219(44.06%)	57(42.54%)	47(47.96%)	15(31.25%)	100(46.08%)	
**Annual household income**						
≤$1025	230(46.28%)	62(46.27%)	51(52.04%)	23(47.92%)	94(43.32%)	4.677
$1026-$12375	258(51.91%)	69(51.49%)	44(44.90%)	24(50.00%)	121(55.76%)	
>$12375	9(1.81%)	3(2.24%)	3(3.06%)	1(2.08%)	2(0.92%)	
**Family history of heart disease**						
Yes	353(71.03%)	99(73.88%)	66(67.35%)	33(68.75%)	155(71.43%)	1.313
No	144(28.97%)	35(26.12%)	32(32.65%)	15(31.25%)	62(28.57%)	
**Tobacco use**						
No	191(38.43%)	43(32.09%)	41(41.84%)	13(27.08%)	94(43.32%)	7.560
Yes	306(61.57%)	91(67.91%)	57(58.16%)	35(72.92%)	123(56.68%)	
**Alcohol use**						
No	315(63.38%)	92(68.66%)	63(64.29%)	29(60.42%)	131(60.37%)	2.672
Yes	182(36.62%)	42(31.34%)	35(35.71%)	19(39.58%)	86(39.63%)	
**Tea habit**						
No	135(27.16%)	102(76.12%)	60(61.22%)	33(68.75%)	135(62.21%)	**8.675***
Yes	82(16.50%)	32(23.88%)	38(38.78%)	15(31.25%)	82(37.79%)	
**Physical exercise**						
No	270(54.32%)	100(74.60%)	41(41.80%)	40(83.30%)	89(41.00%)	**60.193***
Yes	227(45.68%)	34(25.40%)	57(58.20%)	8（16.70%）	128(59.00%)	
**Light diet**						
No	236(47.45%)	95(70.90%)	26(26.50%)	40(83.30%)	75(34.60%)	**85.975***
Yes	261(52.55%)	39(29.10%)	72(73.50%)	8(46.70%)	142(65.40%)	
**T2-T3**						
**Gender**						
Male	280(56.34%)	24(39.34%)	22(47.83%)	67(49.26%)	167(65.75%)	**20.426***

Female	217(43.66%)	37(60.66%)	24(52.17%)	69(50.74%)	87(34.25%)	
**Age**						
<70	259(52.11%)	31(50.80%)	18(39.10%)	39(28.70%)	171(67.30%)	**56.628***
＞=70	238(47.88%)	30(49.20%)	28(60.90%)	97(71.30%)	83(32.70%)	
**Educational attainment**						
High school degree above	322(64.70%)	44(72.10%)	29(63.00%)	95(69.90%)	154(60.60%)	4.958
Other	175(35.20%)	17(27.90%)	17(37.00%)	41(30.10%)	100(39.40%)	
**Career**						
Manual worker	278(55.93%)	30(49.18%)	29(63.04%)	81(59.55%)	138(54.33%)	3.062
Non-manual worker	219(44.06%)	31(50.82%)	17(36.96%)	55(40.44%)	116(45.67%)	
**Annual household income**						
≤$1025	230(46.28%)	23(37.70%)	21(45.65%)	64(47.06%)	122(48.03%)	2.446
$1026-$12375	258(51.91%)	37(60.66%)	24(52.17%)	69(50.74%)	128(50.39%)	
>$12375	9(1.81%)	1(1.64%)	1(2.17%)	3(2.21%)	4(1.57%)	
**Family history of heart disease**						
Yes	353(71.03%)	46(75.41%)	32(69.57%)	100(73.53%)	175(68.90%)	1.591
No	144(28.97%)	15(24.59%)	14(30.43%)	36(26.47%)	79(31.10%)	
**Tobacco use**						
No	200(40.24%)	19(31.15%)	24(52.17%)	41(30.15%)	116(45.67%)	**13.696***
Yes	297(59.76%)	42(68.85%)	22(47.83%)	95(69.85%)	138(54.33%)	
**Alcohol use**						
No	339(68.21%)	46(75.41%)	41(89.13%)	98(72.06%)	154(60.63%)	**18.402***
Yes	158(31.79%)	15(24.59%)	5(10.87%)	38(27.94%)	100(39.37%)	
**Tea habit**						
No	327(65.79%)	48(78.69%)	29(63.04%)	109(80.15%)	141(55.51%)	**29.043***
Yes	170(34.21%)	13(21.31%)	17(36.96%)	27(19.85%)	113(44.49%)	
**Physical exercise**						
No	223(44.90%)	39(63.90%)	8(17.40%)	99(72.80%)	77(30.30%)	**87.627***

Yes	274(55.10%)	22(36.10%)	38(82.60%)	37(27.20%)	177(69.70%)	
**Light diet**						
No	245(49.33%)	42(68.90%)	14(30.40%)	92(67.60%)	97(38.20%)	**46.741***
Yes	252(50.67%)	19(31.10%)	32(69.60%)	44(32.40%)	157(61.80%)	

Note: *Represents p<0.05, the difference between groups is statistically significant.

From Table S1, the PRO physical state transition impact statistically significant covariates as age, gender, tobacco use, alcohol use, tea habit, physical exercise and light diet.

Table S2. The transfer rate of latent transfers under different genders in the physical domain between three time points

	Male		Female	
	Low level	High level	Low level	High level
T1 transfer to T2				
Low level	0.272	0.333	0.342	0.570
High level	0.726	0.667	0.658	0.430
T2 transfer to T3				
Low level	0.752	0.125	0.741	0.298
High level	0.248	0.875	0.259	0.702

Note: Rows for reference time.

Table S3. The transfer rate of latent transfers under different age in the physical domain between three time points

	<70		>=70	
	Low level	High level	Low level	High level
T1 transfer to T2				
Low level	0.180	0.232	0.435	0.575
High level	0.820	0.768	0.565	0.425
T2 transfer to T3				
Low level	0.684	0.153	0.776	0.265
High level	0.316	0.847	0.843	0.735

Note: Rows for reference time.

Table S4. The transfer rate of latent transfers under different tea habit in the physical domain between three time points

	No		Yes	
	Low level	High level	Low level	High level
T1 transfer to T2				
Low level	0.354	0.430	0.283	0.280
High level	0.646	0.570	0.717	0.720
T2 transfer to T3				
Low level	0.789	0.253	0.613	0.103
High level	0.252	0.770	0.308	0.893

Note: Rows for reference time.

Table S5. The transfer rate of latent transfers under different alcohol use in the physical domain between three time points

	No		Yes	
	Low level	High level	Low level	High level
T1 transfer to T2				
Low level	0.240	0.313	0.380	0.425
High level	0.760	0.787	0.620	0.575
T2 transfer to T3				
Low level	0.630	0.140	0.811	0.233
High level	0.370	0.850	0.289	0.767

Note: Rows for reference time.

Table S6. The transfer rate of latent transfers under different tobacco using in the physical domain between three time points

	No		Yes	
	Low level	High level	Low level	High level
T1 transfer to T2				

Low level	0.301	0.288	0.377	0.311
High level	0.699	0.712	0.623	0.687
T2 transfer to T3				
Low level	0.705	0.232	0.883	0.128
High level	0.295	0.768	0.117	0.872

Note: Rows for reference time.

Table S7. The transfer rate of latent transfers under different physical exercise in the physical domain between three time points

	No		Yes	
	Low level	High level	Low level	High level
T1 transfer to T2				
Low level	0.493	0.529	0.123	0.209
High level	0.507	0.471	0.877	0.791
T2 transfer to T3				
Low level	0.925	0.336	0.493	0.110
High level	0.075	0.664	0.507	0.890

Note: Rows for reference time.

Table S8. The transfer rate of latent transfers under different light diet in the physical domain between three time points

	No		Yes	
	Low level	High level	Low level	High level
T1 transfer to T2				
Low level	0.484	0.540	0.137	0.229
High level	0.516	0.460	0.863	0.771
T2 transfer to T3				
Low level	0.842	0.290	0.608	0.114
High level	0.158	0.710	0.392	0.886

Note: Rows for reference time.

Part II: Psychological domain

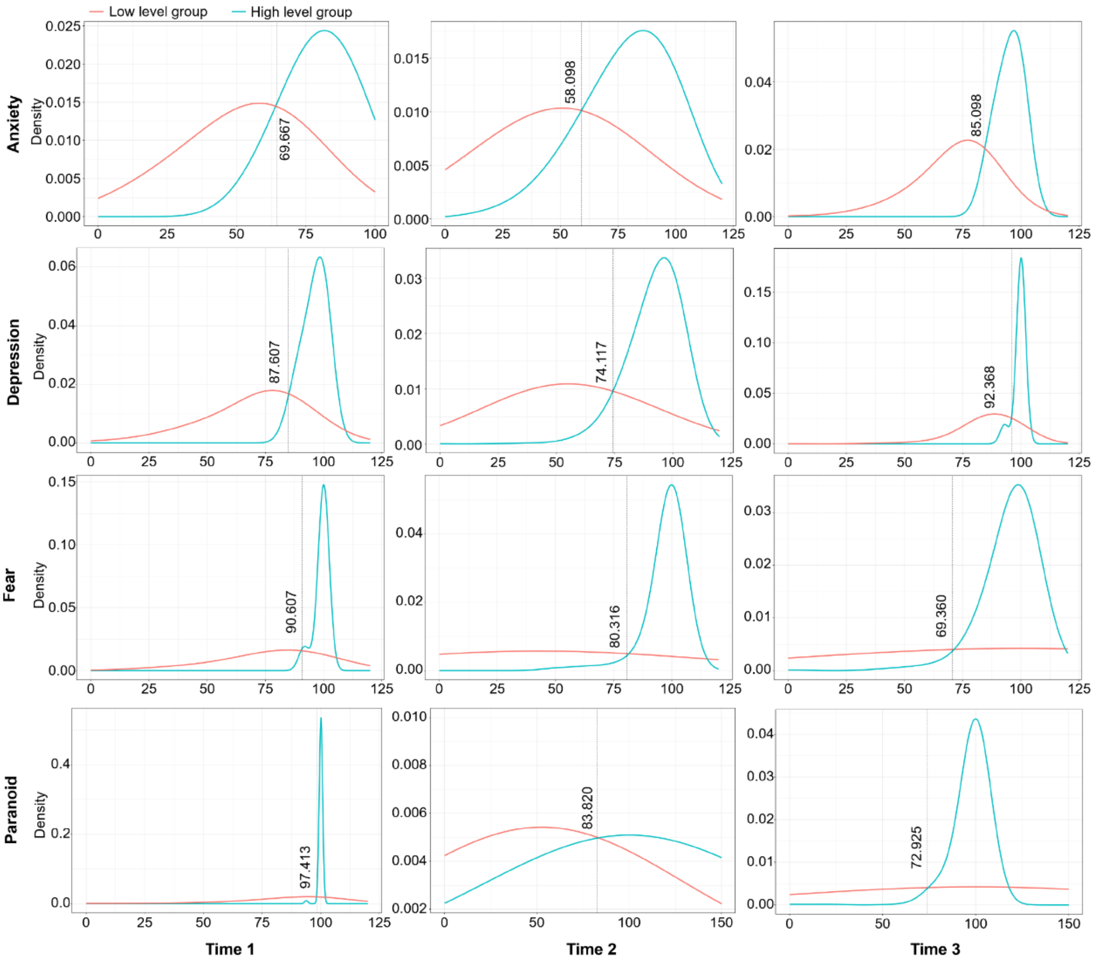

Figure S2. kernel density curves of two potential classes in the psychological domain

Table S9. Latent transfer distribution of covariates in the psychological domain

Covariates	N (%)	Deteriorate	Improve	Low level persistence	High level persistence	χ^2
**T1-T2**						
**Gender**						
Male	280(56.34%)	1(16.67%)	47(46.53%)	16(38.10%)	216(62.07%)	**18.114***
Female	217(43.66%)	5(83.33%)	54(53.47%)	26(61.90%)	132(37.93%)	
**Age**						

<70	259(52.11%)	3(50.00%)	50(49.50%)	20(47.60%)	186(53.40%)	0.875
＞=70	238(47.88%)	3(50.00%)	51(50.50%)	22(52.40%)	162(46.60%)	
**Educational attainment**						
High school degree above	322(64.70%)	4(66.67%)	75(74.30%)	29(69.05%)	214(61.50%)	5.968
Other	175(35.20%)	2(33.34%)	26(25.70%)	13(30.95%)	134(38.50%)	
**Career**						
Manual worker	278(55.93%)	4(66.67%)	69(68.32%)	27(64.29%)	178(51.49%)	**10.987***
Non-manual worker	219(44.06%)	2(33.33%)	32(31.68%)	15(35.71%)	170(48.51%)	
**Annual household income**						
≤$1025	230(46.28%)	3(50.00%)	50(49.50%)	24(57.14%)	153(43.97%)	5.514
$1026-$12375	258(51.91%)	3(50.00%)	51(50.50%)	17(40.48%)	187(53.74%)	
>$12375	9(1.81%)	0(0.00%)	0(0.00%)	1(2.38%)	8(2.30%)	
**Family history of heart disease**						
Yes	353(71.03%)	4(66.67%)	70(69.31%)	29(69.05%)	250(71.84%)	0.392
No	144(28.97%)	2(33.33%)	31(30.69%)	13(30.95%)	98(28.16%)	
**Tobacco use**						
No	191(38.43%)	0(0.00%)	36(35.64%)	10(23.81%)	145(41.67%)	**9.411***
Yes	306(61.57%)	6(100.00%)	65(64.36%)	32(76.19%)	203(58.33%)	
**Alcohol use**						
No	315(63.38%)	4(66.67%)	72(71.29%)	22(52.38%)	217(62.36%)	5.095
Yes	182(36.62%)	2(33.33%)	29(28.71%)	20(47.62%)	131(37.64%)	
**Tea habit**						
No	330(66.40%)	4(66.67%)	70(69.31%)	31(73.81%)	225(64.66%)	1.891
Yes	167(33.60%)	2(33.33%)	31(30.69%)	11(26.19%)	123(35.34%)	
**Physical exercise**						
No	270(54.32%)	2(33.30%)	56(55.40%)	32(76.20%)	180(51.70%)	**10.158***
Yes	227(45.68%)	4(66.70%)	45(44.60%)	10(23.80%)	168(48.30%)	
**Light diet**						

No	236(47.45%)	5(83.30%)	51(50.50%)	23(54.80%)	157(45.10%)	5.135
Yes	261(52.55%)	1(16.70%)	50(49.50%)	19(45.00%)	191(54.90%)	
**T2-T3**						
**Gender**						
Male	280(56.34%)	24(61.54%)	7(31.82%)	10(38.46%)	239(58.29%)	**9.821***
Female	217(43.66%)	15(38.46%)	15(68.18%)	16(61.54%)	171(41.71%)	
**Age**						
<70	259(52.11%)	20(51.30%)	11(50.00%)	12(46.20%)	216(52.70%)	0.473
＞=70	238(47.88%)	19(48.70%)	11(50.00%)	14(53.80%)	194(47.30%)	
**Educational attainment**						
High school degree above	322(64.70%)	26(66.67%)	16(72.70%)	17(65.38%)	263(64.10%)	0.746
Other	175(35.20%)	13(33.33%)	6(27.30%)	9(34.62%)	147(35.90%)	
**Career**						
Manual worker	278(55.93%)	20(51.28%)	16(72.73%)	15(57.69%)	227(55.37%)	2.946
Non-manual worker	219(44.06%)	19(48.72%)	6(27.27%)	11(42.31%)	183(44.63%)	
**Annual household income**						
≤$1025	230(46.28%)	18(46.15%)	12(54.55%)	15(57.69%)	185(45.12%)	4.112
$1026-$12375	258(51.91%)	21(53.85%)	10(45.45%)	10(38.46%)	217(52.93%)	
>$12375	9(1.81%)	0(0.00%)	0(0.00%)	1(3.85%)	8(1.95%)	
**Family history of heart disease**						
Yes	353(71.03%)	27(69.23%)	14(63.64%)	19(73.08%)	293(71.46%)	0.736
No	144(28.97%)	12(30.77%)	8(36.36%)	7(26.92%)	117(28.54%)	
**Tobacco use**						
No	200(40.24%)	18(46.15%)	7(31.82%)	7(26.92%)	168(40.98%)	3.226
Yes	297(59.76%)	21(53.85%)	15(68.18%)	19(73.08%)	242(59.02%)	
**Alcohol use**						
No	339(68.21%)	26(66.67%)	21(95.45%)	25(96.15%)	267(65.12%)	**18.739***
Yes	158(31.79%)	13(33.33%)	1(4.55%)	1(3.85%)	143(34.88%)	

**Tea habit**						
No	327(65.79%)	26(66.67%)	11(50.00%)	16(61.54%)	274(66.83%)	2.856
Yes	170(34.21%)	13(33.33%)	11(50.00%)	10(38.46%)	136(33.17%)	
**Physical exercise**						
No	223(44.90%)	30(76.90%)	7(31.80%)	17(65.40%)	169(41.20%)	**24.345***
Yes	274(55.10%)	9(23.10%)	15(68.20%)	9(34.60%)	241(58.80%)	
**Light diet**						
No	245(49.33%)	22(56.40%)	11(50.00%)	14(53.80%)	198(48.30%)	1.175
Yes	252(50.67%)	17(43.60%)	11(50.00%)	12(46.20%)	212(51.70%)	

Note: *Represents p<0.05, the difference between groups is statistically significant.

From Table S9 the PRO physiological state transition impact statistically significant covariates as gender, career, tobacco use, alcohol use and physical exercise.

Table S10. The transfer rate of latent transfers under different genders in the physiological domain between three time points

	Male		Female	
	Low level	High level	Low level	High level
T1 transfer to T2				
Low level	0.253	0.004	0.325	0.036
High level	0.747	0.996	0.675	0.964
T2 transfer to T3				
Low level	0.588	0.091	0.516	0.080
High level	0.412	0.909	0.484	0.919

Note: Rows for reference time.

Table S11. The transfer rate of latent transfers under different career in the physiological domain between three time points

	Manual worker		Non-manual worker	
	Low level	High level	Low level	High level
T1 transfer to T2				

Low level	0.281	0.021	0.319	0.011
High level	0.719	0.979	0.681	0.989
T2 transfer to T3				
Low level	0.483	0.080	0.647	0.094
High level	0.517	0.920	0.353	0.906

Note: Rows for reference time.

Table S12. The transfer rate of latent transfers under different tobacco using in the physiological domain between three time points

	No		Yes	
	Low level	High level	Low level	High level
T1 transfer to T2				
Low level	0.217	0.005	0.329	0.028
High level	0.783	0.995	0.671	0.972
T2 transfer to T3				
Low level	0.500	0.096	0.558	0.079
High level	0.500	0.904	0.442	0.921

Note: Rows for reference time.

Table S13. The transfer rate of latent transfers under different alcohol use in the physiological domain between three time points

	No		Yes	
	Low level	High level	Low level	High level
T1 transfer to T2				
Low level	0.575	0.089	0.325	0.007
High level	0.425	0.911	0.675	0.993
T2 transfer to T3				
Low level	0.543	0.088	0.500	0.083
High level	0.457	0.912	0.500	0.917

Note: Rows for reference time.

Table S14. The transfer rate of latent transfers under different physical exercise in the physiological domain between three time points

	No		Yes	
	Low level	High level	Low level	High level
T1 transfer to T2				
Low level	0.363	0.010	0.181	0.023
High level	0.634	0.990	0.819	0.977
T2 transfer to T3				
Low level	0.708	0.150	0.375	0.036
High level	0.292	0.850	0.625	0.964

Note: Rows for reference time.

Part III: Social domain

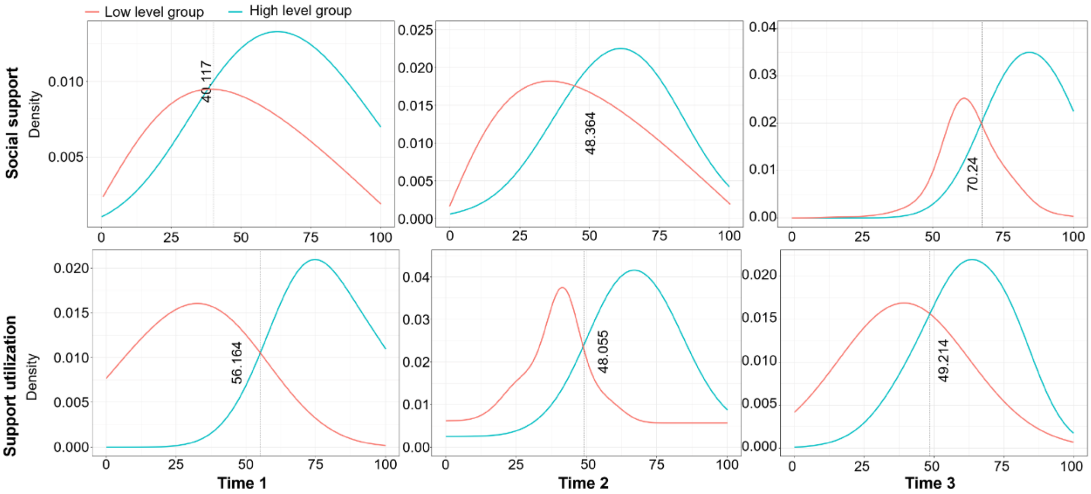

Figure S3. kernel density curves of two potential classes in the social domain

Table S15. Latent transfer distribution of covariates in the social domain

Covariates	N (%)	Deteriorate	Improve	Low level persistence	High level persistence	χ^2
**T1-T2**						
**Gender**						
Male	280(56.34%)	40(63.49%)	53(51.96%)	85(58.22%)	102(54.84%)	2.485
Female	217(43.66%)	23(36.51%)	49(48.04%)	61(41.78%)	84(45.16%)	
**Age**						
<70	259(52.11%)	42(66.70%)	46(45.10%)	70(47.90%)	101(54.30%)	**8.732***
＞=70	238(47.88%)	21(33.30%)	56(54.90%)	76(52.10%)	85(45.70%)	
**Educational attainment**						
High school degree above	322(64.70%)	42(66.67%)	63(61.80%)	103(70.50%)	114(61.30%)	3.627
Other	175(35.20%)	21(33.33%)	39(38.20%)	43(29.50%)	72(38.70%)	
**Career**						
Manual worker	278(55.93%)	35(55.56%)	56(54.90%)	77(52.74%)	110(59.13%)	1.428
Non-manual worker	219(44.06%)	28(44.44%)	46(45.10%)	69(47.26%)	76(40.87%)	

**Annual household income**						
≤$1025	230(46.28%)	34(53.97%)	41(40.20%)	69(47.26%)	86(46.24%)	4.214
$1026-$12375	258(51.91%)	28(44.44%)	58(56.86%)	74(50.68%)	98(52.69%)	
>$12375	9(1.81%)	1(1.59%)	3(2.94%)	3(2.05%)	2(1.08%)	
**Family history of heart disease**						
Yes	353(71.03%)	45(71.43%)	73(71.57%)	101(69.18%)	134(72.04%)	0.355
No	144(28.97%)	18(28.57%)	29(28.43%)	45(30.82%)	52(27.96%)	
**Tobacco use**						
No	191(38.43%)	24(38.10%)	37(36.27%)	57(39.04%)	73(39.25%)	0.279
Yes	306(61.57%)	39(61.90%)	65(63.73%)	89(60.96%)	113(60.75%)	
**Alcohol use**						
No	315(63.38%)	39(61.90%)	65(63.73%)	93(63.70%)	118(63.44%)	0.071
Yes	182(36.62%)	24(38.10%)	37(36.27%)	53(36.30%)	68(36.56%)	
**Tea habit**						
No	135(27.16%)	39(61.90%)	69(67.65%)	100(68.49%)	122(65.59%)	0.983
Yes	82(16.50%)	24(38.10%)	33(32.35%)	46(31.51%)	64(34.41%)	
**Physical exercise**						
No	270(54.32%)	37(58.70%)	55(53.90%)	81(55.50%)	97(52.20%)	0.932
Yes	227(45.68%)	24(41.30%)	47(46.10%)	65(44.50%)	89(47.80%)	
**Light diet**						
No	236(47.45%)	160(49.10%)	36(42.90%)	27(39.70%)	13(68.40%)	6.044
Yes	261(52.55%)	166(50.90%)	48(57.10%)	41(60.30%)	6（31.60%）	
**T2-T3**						
**Gender**						
Male	280(56.34%)	118(53.64%)	33(66.00%)	92(57.86%)	37(54.41%)	2.803
Female	217(43.66%)	102(46.36%)	17(34.00%)	67(42.14%)	31(45.59%)	
**Age**						
<70	259(52.11%)	113(51.40%)	25(50.00%)	87(54.70%)	34(50.00%)	0.693
＞=70	238(47.88%)	107(48.60%)	25(50.00%)	72(45.30%)	34(50.00%)	

**Educational attainment**						
High school degree above	322(64.70%)	130(59.10%)	31(62.00%)	114(71.70%)	47(69.12%)	7.187
Other	175(35.20%)	90 (40.90%)	19(19.00%)	45(28.30%)	21(30.88%)	
**Career**						
Manual worker	278(55.93%)	130(59.09%)	23(46.00%)	89(55.97%)	36(52.94%)	3.139
Non-manual worker	219(44.06%)	90(40.91%)	27(54.00%)	70(44.03%)	32(47.06%)	
**Annual household income**						
≤$1025	230(46.28%)	99(45.00%)	23(46.00%)	80(50.31%)	28(41.18%)	3.519
$1026-$12375	258(51.91%)	117(53.18%)	25(50.00%)	77(48.43%)	39(57.35%)	
>$12375	9(1.81%)	4(1.82%)	2(4.00%)	2(1.26%)	1(1.47%)	
**Family history of heart disease**						
Yes	353(71.03%)	156(70.91%)	32(64.00%)	114(71.70%)	51(75.00%)	1.758
No	144(28.97%)	64(29.09%)	18(36.00%)	45(28.30%)	17(25.00%)	
**Tobacco use**						
No	200(40.24%)	91(41.36%)	20(40.00%)	65(40.88%)	24(35.29%)	0.836
Yes	297(59.76%)	129(58.64%)	30(60.00%)	94(59.12%)	44(64.71%)	
**Alcohol use**						
No	339(68.21%)	152(69.09%)	32(64.00%)	108(67.92%)	47(69.12%)	0.519
Yes	158(31.79%)	68(30.91%)	18(36.00%)	51(32.08%)	21(30.88%)	
**Tea habit**						
No	327(65.79%)	146(66.36%)	35(70.00%)	109(68.55%)	37(54.41%)	4.877
Yes	170(34.21%)	74(33.64%)	15(30.00%)	50(31.45%)	31(45.59%)	
**Physical exercise**						
No	223(44.90%)	94(42.70%)	22(44.00%)	80(50.30%)	27(39.70%)	3.062
Yes	274(55.10%)	126(57.30%)	28(56.00%)	79(49.70%)	41(60.30%)	
**Light diet**						
No	245(49.33%)	112(50.90%)	18(36.00%)	81(50.90%)	34(50.00%)	3.952
Yes	252(50.67%)	108(49.10)	32(64.00%)	78(49.10%)	34(50.00%)	

Note: *Represents $p<0.05$, the difference between groups is statistically significant.

From Table S15. the PRO social state transition impact statistically significant covariates as

age.

Table S16. The transfer rate of latent transfers under different age in the social domain between three time points

	<70		>=70	
	Low level	High level	Low level	High level
T1 transfer to T2				
Low level	0.603	0.293	0.575	0.198
High level	0.397	0.707	0.425	0.802
T2 transfer to T3				
Low level	0.776	0.768	0.742	0.758
High level	0.224	0.232	0.258	0.242

Note: Rows for reference time.

Part IV: Therapeutic domain

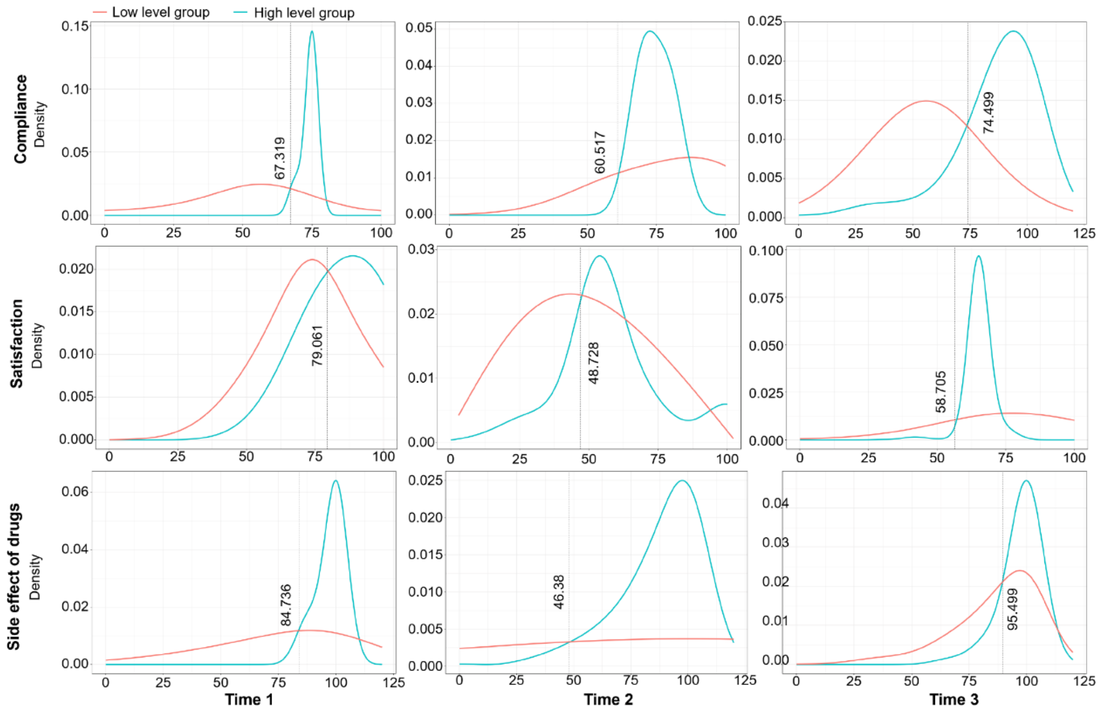

Figure S4. kernel density curves of two potential classes in the therapeutic domain

Table S17. Latent transfer distribution of covariates in the therapeutic domain

Covariates	N (%)	Deteriorate	Improve	Low level persistence	High level persistence	χ^2
**T1-T2**						
**Gender**						
Male	280(56.34%)	192(58.90%)	49(58.33%)	30(44.21%)	9(47.37%)	5.753
Female	217(43.66%)	134(41.10%)	35(41.67%)	38(55.21%)	10(52.63%)	
**Age**						
<70	259(52.11%)	172(52.80%)	44(52.40%)	34(50.00%)	9(47.40%)	0.350
＞=70	238(47.88%)	154(47.20%)	40(47.60%)	34(50.00%)	10(52.60%)	
**Educational attainment**						
High school degree above	322(64.70%)	230(70.55%)	36(42.90%)	45(66.32%)	11(57.89%)	**22.911***

Other	175(35.20%)	96(29.45%)	48(57.10%)	23(33.19%)	8(42.11%)	
**Career**						
Manual worker	278(55.93%)	181(55.52%)	48(57.14%)	39(57.21%)	10(52.63%)	0.212
Non-manual worker	219(44.06%)	145(44.48%)	36(42.86%)	29(42.21%)	9(47.37%)	
**Annual household income**						
≤$1025	230(46.28%)	172(52.76%)	11(13.10%)	40(58.21%)	7(36.84%)	**48.453***
$1026-$12375	258(51.91%)	148(45.40%)	71(84.52%)	27(39.21%)	12(63.16%)	
>$12375	9(1.81%)	6(1.84%)	2(2.38%)	1(1.96%)	0(0.00%)	
**Family history of heart disease**						
Yes	353(71.03%)	236(72.39%)	57(67.86%)	43(63.32%)	17(89.47%)	5.853
No	144(28.97%)	90(27.61%)	27(32.14%)	25(36.19%)	2(10.53%)	
**Tobacco use**						
No	191(38.43%)	136(41.72%)	27(32.14%)	21(30.11%)	7(36.84%)	4.550
Yes	306(61.57%)	190(58.28%)	57(67.86%)	47(69.31%)	12(63.16%)	
**Alcohol use**						
No	315(63.38%)	202(61.96%)	54(64.29%)	46(67.32%)	13(68.42%)	1.053
Yes	182(36.62%)	124(38.04%)	30(35.71%)	22(32.11%)	6(31.58%)	
**Tea habit**						
No	135(27.16%)	215(65.95%)	56(66.67%)	48(70.12%)	11(57.89%)	1.183
Yes	82(16.50%)	111(34.05%)	28(33.33%)	20(29.81%)	8(42.11%)	
**Physical exercise**						
No	270(54.32%)	187(57.40%)	38(45.20%)	32(47.10%)	13(68.40%)	6.076
Yes	227(45.68%)	139(42.60%)	46(54.80%)	36(52.90%)	6（31.60%）	
**Light diet**						
No	236(47.45%)	160(49.10%)	36(42.90%)	27(39.70%)	13(68.40%)	6.044
Yes	261(52.55%)	166(50.90%)	48(57.10%)	41(60.30%)	6（31.60%）	

**T2-T3**						
**Gender**						
Male	280(56.34%)	36(54.55%)	52(57.78%)	170(55.92%)	22(59.64%)	0.330
Female	217(43.66%)	30(45.45%)	38(42.22%)	134(44.08%)	15(40.36%)	
**Age**						
<70	259(52.11%)	32(48.50%)	38(42.20%)	168(55.30%)	21(56.80%)	5.405
＞=70	238(47.88%)	34(51.50%)	52(57.80%)	136(44.70%)	16(43.20%)	
**Educational attainment**						
High school degree above	322(64.70%)	33(50.00%)	62(68.89%)	213(70.07%)	14(37.80%)	**22.482***
Other	175(35.20%)	33(50.00%)	28(31.11%)	91(29.93%)	23(62.20%)	
**Career**						
Manual worker	278(55.93%)	31(46.97%)	53(58.89%)	167(54.93%)	27(72.97%)	6.952
Non-manual worker	219(44.06%)	35(53.03%)	37(41.11%)	137(45.07%)	10(27.02%)	
**Annual household income**						
≤$1025	230(46.28%)	16(24.24%)	42(46.67%)	170(55.92%)	2(5.68%)	**52.407***
$1026-$12375	258(51.91%)	49(74.24%)	48(53.33%)	127(41.78%)	34(91.11%)	
>$12375	9(1.81%)	1(1.52%)	0(0.00%)	7(2.30%)	1(2.70%)	
**Family history of heart disease**						
Yes	353(71.03%)	44(66.67%)	55(61.11%)	224(73.68%)	30(81.13%)	7.770
No	144(28.97%)	22(33.33%)	35(38.89%)	80(26.32%)	7(18.87%)	
**Tobacco use**						
No	200(40.24%)	24(36.36%)	39(43.33%)	125(41.12%)	12(32.04%)	1.806
Yes	297(59.76%)	42(63.64%)	51(56.67%)	179(58.88%)	25(67.96%)	
**Alcohol use**						
No	339(68.21%)	48(72.73%)	61(67.78%)	203(66.78%)	27(72.81%)	1.304
Yes	158(31.79%)	18(27.27%)	29(32.22%)	101(33.22%)	10(27.19%)	
**Tea habit**						
No	327(65.79%)	39(59.09%)	59(65.56%)	208(68.42%)	21(56.59%)	3.595

Yes	170(34.21%)	27(40.91%)	31(34.44%)	96(31.58%)	16(43.41%)	
**Physical exercise**						
No	223(44.90%)	36(54.50%)	32(35.60%)	142(46.70%)	13(35.10%)	7.488
Yes	274(55.10%)	30(45.50%)	58(64.40%)	162(53.30%)	24(64.90%)	
**Light diet**						
No	245(49.33%)	28(42.40%)	41(45.60%)	161(53.00%)	15(40.50%)	5.187
Yes	252(50.67%)	38(57.60%)	49(54.40%)	143(47.00%)	22(59.50%)	

Note: *Represents p<0.05, the difference between groups is statistically significant.

From Table S17. the PRO therapeutic state transition impact statistically significant covariates as educational attainment and annual household income.

Table S18. The transfer rate of latent transfers under different educational attainment in the physical domain between three time points

	Other		High school degree above	
	Low level	High level	Low level	High level
T1 transfer to T2				
Low level	0.555	0.954	0.323	0.923
High level	0.445	0.046	0.677	0.077
T2 transfer to T3				
Low level	0.774	0.702	0.764	0.589
High level	0.226	0.298	0.236	0.411

Note: Rows for reference time.

Table S19. The transfer rate of latent transfers under different annual household income in the physical domain between three time points

	low-income group		middle- and high-income group	
	Low level	High level	Low level	High level
T1 transfer to T2				
Low level	0.784	0.960	0.277	0.927
High level	0.216	0.040	0.723	0.073

T2 transfer to T3				
Low level	0.801	0.888	0.736	0.588
High level	0.199	0.112	0.264	0.412

Note: Rows for reference time.

Reference

1. Han Q, Ren J, Tian J, et al. A nomogram based on a patient-reported outcomes measure: predicting the risk of readmission for patients with chronic heart failure[J]. Health and Quality of Life Outcomes, 2020, 18(1): 1-8.
2. Zakharova E, Gaipov A, Bello A K, et al. International Society of Nephrology Global Kidney Health Atlas: structures, organization, and services for the management of kidney failure in Newly Independent States and Russia[J]. Kidney International Supplements, 2021, 11(2): e57-e65.
3. Murphy M H, Blair S N, Murtagh E M. Accumulated versus continuous exercise for health benefit[J]. Sports medicine, 2009, 39(1): 29-43.
4. Colin-Ramirez E, Ezekowitz J A. Salt in the diet in patients with heart failure: what to recommend[J]. Current opinion in cardiology, 2016, 31(2): 196-203.
